# The Diabetes App Challenge: User-Led Development and Piloting of Internet Applications Enabling Young People With Diabetes to Set the Focus for Their Diabetes Consultations

**DOI:** 10.2196/med20.3032

**Published:** 2014-11-07

**Authors:** Emily J Ashurst, Ray B Jones, Charles Abraham, Martin Jenner, Kate Boddy, Rachel EJ Besser, Suzanne Hammersley, Jonathan Pinkney

**Affiliations:** ^1^Plymouth UniversitySchool of Nursing and MidwiferyPlymouthUnited Kingdom; ^2^Exeter Medical SchoolUniversity of ExeterExeterUnited Kingdom; ^3^Diabetes UKBarnstableUnited Kingdom; ^4^Plymouth University Peninsula Medical SchoolPlymouth UniversityPlymouthUnited Kingdom

**Keywords:** Type 1 diabetes, adolescents, mobile technology, clinic appointment, user-innovation, self-care, user-centered design

## Abstract

**Background:**

Traditionally, some teenagers and young adults with diabetes have not engaged well at diabetes appointments, giving rise to concerns about long-term health risks. We considered that apps might help this group of patients to improve preparation for, and therefore engagement at their appointments. Although there are already many apps for young people with type 1 diabetes (YPD), we thought that by supporting YPD themselves to develop apps, the resulting products would have greater “authenticity” and relevance.

**Objective:**

To test the feasibility of an online competition to (1) recruit and support YPD to develop apps (mobile or Internet based) to help prepare for clinic appointments, and (2) for these apps to be tested and rated by YPD.

**Methods:**

The “Diabetes App Challenge” was a United Kingdom (UK) national competition, run between June and October 2012 for teams including at least one YPD (aged 16-25) to pilot the design and development of apps for use by other YPD prior to clinic appointments. The competition was advertised by social media, email, AdWords and postings on the Diabetes UK website. Registrants for the competition were supported via email and discussion forum. After app development, other YPD were invited (November 2012-February 2013) to trial the apps, choose and use one prior to a clinic appointment, and review their experiences.

**Results:**

Of 56 people (including 28 YPD) who expressed interest in the competition, 6 teams (14 people) developed and submitted an app. Two apps aimed to facilitate agenda setting in clinic consultations, 2 enabled data logging and 2 helped insulin dose calculation. Of 135 YPD who registered to trial the apps, 83 (61.5%) took part (mean age 18.98, 37/83 male). Agenda setting apps were considered most useful for preparing for and setting the focus of clinic appointments (*P*=.02). Just over half (46/83, 55%) said they would use their chosen app again and 4/5 (67/83, 81%) would recommend it to a friend.

**Conclusions:**

This competition to engage YPD in developing and reviewing apps proved successful. App designers and testers saw a need for a range of functions. However, this may, in part, reflect a lack of detailed knowledge of all existing apps and be limited by the technical skills of YPD. App competitions appear worth applying to other patient groups, but future competitions should include a review stage and perhaps focus on ideas for app design for subsequent professional implementation.

## Introduction

There is widespread interest in harnessing the potential of apps to promote better self-care of young people with type 1 diabetes (YPD). However, websites and apps are likely to be most popular if they really engage and interest patients in terms of both style and content [1]. Programmers working with user representatives are likely to optimize such recipient-app “fit” but differences of language, attitudes, and values may remain a problem [2], and programmer-led rather than patient-led app development may inhibit innovation. Problems may be minimized if developers themselves are drawn from user communities; mobile “platforms” are becoming easier allowing users with limited skills to customize and create their own innovative designs [3]. This approach has been tried before in user-led competitions inviting user submitted designs to be trialled and reviewed by other users [4-7] but not with YPD.

Many YPD demonstrate poor blood glucose control [8], which if sustained, is the strongest risk factor for the development of future complications [9] and reduced life expectancy [10]. Helping YPD engage with health services, to manage their condition and achieve better diabetes control is essential [11]. Health care professionals need to connect with a wide range of differently-motivated YPD to help them cope with diabetes and achieve optimal diabetes control [12]. It is thought that a stronger emphasis is needed on “patient-centered” consultations for YPD [13], and finding innovative ways to enhance the active involvement of YPD in agenda-setting in diabetes consultations and engaged self-management [14]. User-centered apps that may improve blood glucose monitoring have been piloted [15], yet to our knowledge, although there is (locally) current pilot work on web-based pre-clinic agenda setting for adults with diabetes [16], there were no apps aimed at YPD for consultation engagement.

The objective of this study was to evaluate a new way to engage YPD in designing and producing apps to improve engagement at diabetes clinic appointments. The aim of the Diabetes App Challenge was to test the feasibility of using a UK national online “competition” to (1) recruit and support YPD to design and develop apps to help set the focus for diabetes consultations, and (2) recruiting other YPD to test these apps.

## Methods

### Ethics

Ethical approval for the study was granted (12/SW/0121, 28^th^ May 2012) by the Cornwall & Plymouth National Research Ethics Service Committee of the National Health Service (NHS).

### Design and Sample Size

#### Overview

The study comprised 2 stages: (1) a UK competition in which YPD and teammates (“developers”) developed an app; and (2) YPD (“reviewers”) were invited to test and review the submitted apps. We could not predict how many apps would be submitted but anticipated up to 10 entries and had one app developed before the competition via a student project. This app was available (1) as an example for other developers, and (b) as one entrant for the competition and for review. In stage two, our target was to recruit up to 200 reviewers.

#### Stage 1 Developers

**Figure 1:**
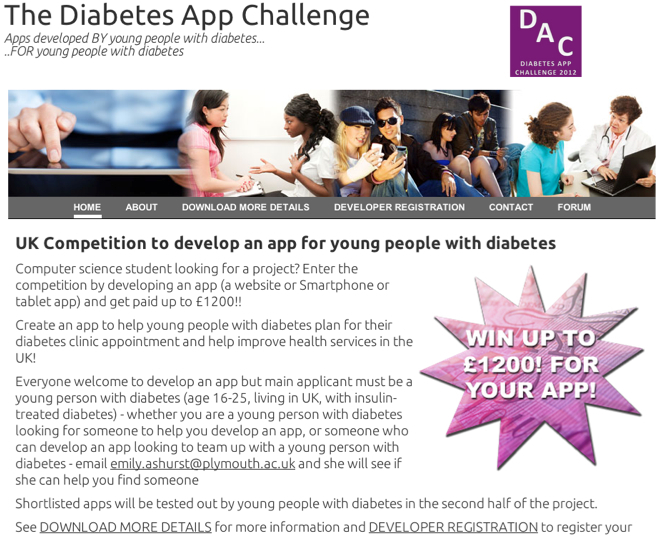
Screenshot of developer stage website homepage.

Developer teams had to include at least one person aged 16-25 with type 1 diabetes living in the UK. Various online methods were used to raise awareness of the competition. Interested parties were directed to the project website for participant information, consent, and registration (Figure 1). Methods included: (1) email to 416 pediatricians and adult diabetes consultants and 160 computer science lecturers of UK universities following online searches for contacts; (2) 68 messages posted on university computer science, students union and diabetes relevant Facebook and Twitter pages; (3) paid advertisements set up via Google AdWords (800 GBP) and Facebook (900 GBP) Campaigns; (4) Diabetes UK postings on their website, Facebook, newsletters, and Balance and Update magazines; (5) project and personal Facebook and Twitter pages of the team, project advisory group, and other supporting members; (6) press releases and website posts by the host Universities; (7) posts in diabetes discussion forums; (8) emails to listserves and contacts of the team.

Following expression of interest by email from YPD without app developing experience, and from app developers without diabetes, an email “match-making” service was offered to facilitate the creation of appropriate teams by the first author (EA). Teams were given links to useful resources, tips and suggestions, and were offered technical support through a website forum.

The developers’ challenge was to create smartphone apps or websites that would be useful in preparing YPD for clinic appointments and help set the focus of the consultation. Applicants were shown the example of the first entrant website You + Your Diabetes [17], created by a YPD as a student project in Plymouth University. This used an agenda setting approach with prompts for topics. Regular emails were sent to teams to ascertain progress, answer queries and signpost the forum for more information, discussion and technical support. Teams submitting apps to the competition were awarded 65 GBP towards their publishing and hosting costs, 100 GBP for maintaining their app over the course of the project, 6 GBP for each reviewer that chose their app from a maximum (target) of 200 reviewers and a certificate of achievement. Advertising and competition ran from June to October 2012.

Submitted apps were reviewed by the project team for suitability and accuracy before offering to YPD reviewers in stage two. Developers maintained and updated their apps through stage two and were able to monitor feedback from reviewers in the forum.

#### Stage 2 Reviewers

The target audience was initially people aged 16-22 with type 1 diabetes living in the UK and with clinic appointments due within the 4 month recruitment period (Mid November – Mid February 2013). However, from mid-December, due to initially lower than expected registrations the upper age limit was extended from 22 to 25.

**Figure 2:**
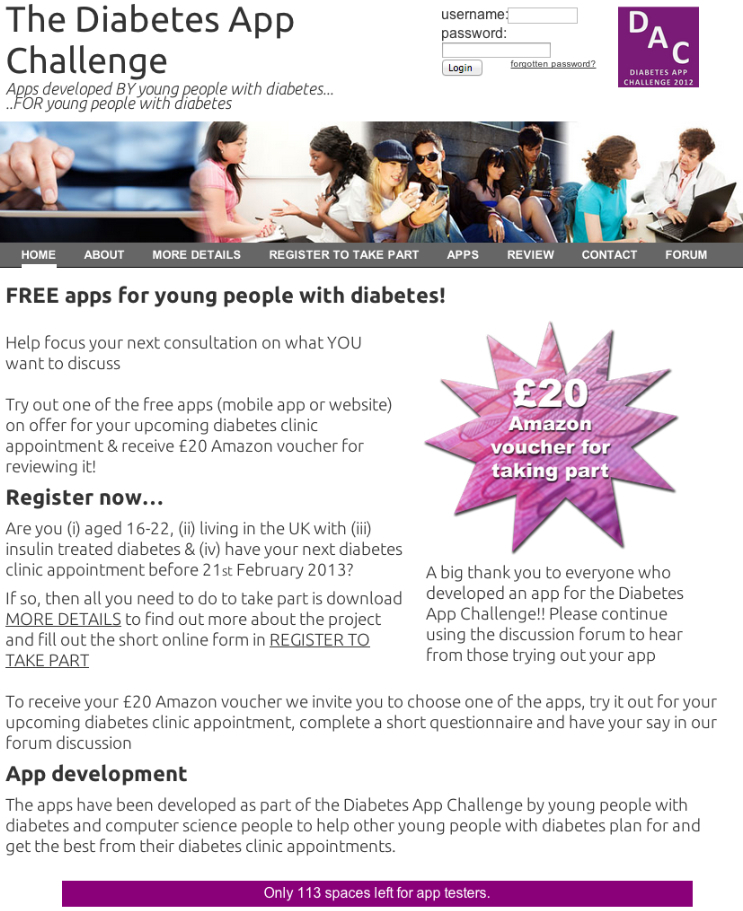
Screenshot of reviewer stage website homepage.

Using similar methods to raise awareness as in stage 1, those previously made aware of the project were contacted with updates of stage 2. In addition, 50 university and 54 GP surgeries were emailed and others contacted by social media. Interested parties were directed to the project website which included information, consent, and registration for potential reviewers (Figure 2).

**Figure 3:**
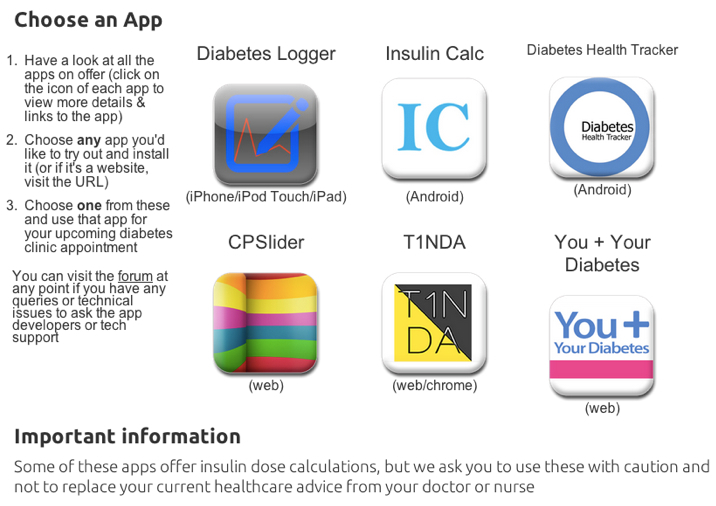
Screenshot of reviewer stage website app selection.

Following completion of a baseline questionnaire, those registered were given login access to the website and discussion forum where links to the apps were located. YPD were asked to (1) examine and try the apps on offer, (2) choose one, and (3) use it in preparation for their upcoming clinic appointment (Figure 3). Registrants were advised that apps were not a substitute for medical advice. After the appointment, they were asked to (4) complete a review and follow-up questionnaire, (5) and add comments in the forum. For successfully completing the review questionnaire and posting a minimum of one post in the discussion forum, reviewers were awarded a 20 GBP Amazon voucher via email.

### Patient and Public Involvement

As well as involving YPD in the development and review of apps, a small group comprising 4 YPD were involved in developing the research project to help ensure it was relevant to other YPD. They helped write and review participant consent forms to ensure accessibility, helped design the Diabetes App Challenge website, created videos to provide further information and were consulted throughout the duration of the project.

### Data Collection

For developers, website registration forms collected demographic data. After participation in the competition, feedback questions regarding competition experience were sent by email and other forum and email communication was collected for content analysis. Those who did not submit an app to the competition (“non-developers”) were followed up by email questionnaire to establish reasons for this and to learn for future competition delivery. For this feedback, non-developers were offered each a 20 GBP Amazon voucher.

For reviewers, the website registration form requested (1) demographic data, (2) next appointment due date, (3) if they had missed or considered missing a clinic appointment and (4) “before-after" questions about satisfaction and confidence (piloting for a subsequent study). After app use, the website follow-up form requested information about the app they used including (1) initial attraction to that app, (2) ease of use, (3) perceived usefulness of the app in preparing for and focusing during their clinic appointment, (4) intentions to use the app again and recommend to a friend, (5) helpfulness of app features, and (6) follow-up questions. Additional follow-up questions via email included (1) importance placed on YPD-created apps and (2) further qualitative feedback on the Diabetes App Challenge experience.

### Analyses

Descriptive responses to open-ended questions (email and online questionnaire) were analyzed using an inductive method of conventional content analysis to identify and summarize response meanings [18], and repeat occurrences of similar meanings between participant responses were counted and identified as reflecting a common issue of importance in summative content analysis [19].

## Results

### Stage 1 Developers

#### Overview

Six teams (6 YPD and 8 teammates) submitted a completed app to the competition comprising two match-made teams, two self-made teams and two teams of one YPD each (including the “initial” student). Teams were located across England. The 6 YPD had a mean age of 20.33 (SD 3.27) and a mean of 8.75 years diabetes duration (SD 7.36) and the 8 teammates had a mean age of 21.50 (SD 2.73). Half of developers, both YPD and teammates, were computer science university students (7/14), 2 were in computer science employment (14%), 4 were students in other subjects (29%) and 1 in unknown employment (7%).

**Figure 4:**
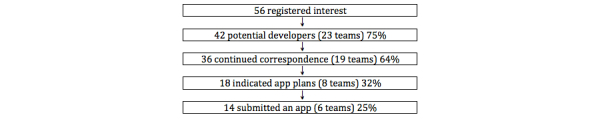
Flow diagram showing developer participant numbers from recruitment through to app submission.

In total 56 people registered interest to develop an app. Excluding enquiries with no further interest and offers of support from people without diabetes or technical skills, there were 23 teams of potential developers. These included 9 match-made teams, 11 teams of one YPD and 3 self-made teams. Nineteen teams continued corresponding with EA and 8 teams described plans to develop an app. Of those, 25% who expressed initial interest submitted apps (Figure 4).

Of the 42 potential developers, a third (15/42) were recruited from emails to clinicians, universities, and others, 7 from Diabetes UK, 4 from Facebook/Twitter, 2 were known contacts of the project, 1 from Google AdWords. A third (13/42) could not be traced to the original advertising source.

Nineteen (68%) of the 28 potential developers who did not submit an app responded to email follow-up. The reasons for not completing an app were lack of time or other commitments (11/19), communication or conflict of ideas within match-made teams (6/19), realization that their design already existed (1/19), and app coding difficulties (1/19). Although incomplete or just ideas, seven made reference to their app plans, including four data-logging, two notes/ agenda setting, and one diabetes game.

**Figure 5:**
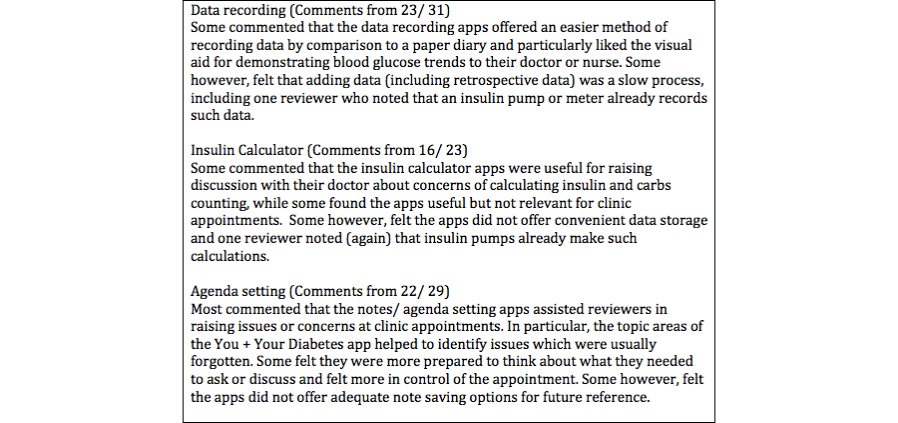
Summary of comments made about apps percieved usefulness (n=61).

#### Submitted Apps

The 6 submitted apps were, Diabetes Logger [20], Diabetes Health Tracker [21], You + Your Diabetes [17], T1NDA [22], Insulin Calc [23], and cpSlider [24]. The main functions of the apps were: (1) recording and viewing data; (2) helping calculate insulin dosage; and (3) making notes/ agenda setting. Of these, 2 were submitted on iOS, 2 for Android and 3 were websites (Table 1).

#### Support and Communication

Across the course of the project EA sent an average of 43 emails to each developer (competition information, requests for progress updates, responses to queries and technical support, updates throughout reviewer stage and payments). Developers sent EA 12 emails each (updates on progress, queries, and feedback regarding the Diabetes App Challenge experience). Two teams requested support regarding formula accuracy of insulin dose calculations, and one team technical support regarding coding. The most common difficulty cited for developers was time, mostly limited by university assignments or employment. Other difficulties included design and technical skills and team communication.

#### Reasons for Creating the Apps

YPD developers took part in the competition to make it easier for others to manage their appointments and condition, as well teammates gained app project experience (Textbox 1).

**Table 1:** Main functions and platforms of submitted apps and number of reviewers who chose each app.

App name	Device	App function			
		Data recording	Insulin dose calculator	Notes/ agenda setting	Reviewers choosing
Diabetes Logger	iOS	✓			25
Diabetes Health Tracker	Android	✓			6
Insulin Calc	Android/ iOS		✓		17
CPSlider	Website		✓		6
T1NDA	Website			✓	5
You + Your Diabetes	Website			✓	24
Reviewers choosing		31	23	29	83

**Reasons for app function design by five YPD developers.**

“I have had problems in the past with showing my BG results to consultants and have had problems recording them”

“there is absolutely no point whatsoever in going to a diabetic clinic appointment at a hospital without an accurate record of blood sugar etc. as there is really very little anyone can do for you without it”

“keeping track of lots of data was a real pain, and having to do so to make the consultations useful, quite time consuming”

“The main reason for me to want to make this app was in the hope that it would help someone with their new diagnosis”

“I was motivated by the challenge of deigning an app for people like myself, who have type 1 in the hope that in some way it would make there life better of easier”

### Stage 2 Reviewers

#### Overview

Of the 135 YPD reviewer registrants, 83 (62%) took part in trialling and reviewing an app. Reviewers’ mean age was 18.98 (SD 2.58), 55% were female, and mean years since diagnosis was 7.08 (SD 5.05) ranging from 2 months to over 20 years. Fifty-one (62%) had attended a clinic appointment within the last 3 months, 27 (33%) within 4-8 months, 4 (5%) over 9 months ago. Twelve (14%) had missed, and 19 (23%) had considered missing clinic appointments at some time because they did not think it was worth attending, while 47 (57%) had never considered it.

Reviewers were made aware of the Diabetes App Challenge through (1) Diabetes UK (22/83), (2) Twitter (14/83), (3) Facebook (13/83), (4) word of mouth from friend or family (6/83), (5) email, (8/83), (6) hospital (5/83), (7) newspaper (4/83), (8) doctor’s surgery/ letter (5/83), (9) online search (4/83), (10) diabetes discussion forum (1/83), and (11) university news (1/83).

#### Reviewer Choice

Reviewers looked at an average of 3 apps each before making a selection (based on self-report and tracking logs). The three most popular apps chosen by reviewers each offered a different function: Diabetes Logger (n=25)m a data recording app; You + Your Diabetes (n=24), a notes/ agenda setting app; and Insulin Calc (n=17), an insulin dose calculator app (Table 1).

#### Perceived Usefulness of App for Preparing for or Setting the Focus of Clinic Appointment

Over half of reviewers (52/83, 63%) thought their chosen app was useful or very useful for preparing for or setting the focus of their clinic appointment. Notes/ agenda setting apps were considered more useful (mean 4.10, SD .77) for clinic appointments than data logging (mean 3.36, SD 1.08) and insulin dose calculator (mean 3.22, SD 1.28) (*F*_2,80_=5.72, *P*=.01). Comments from 61/83 reviewers reflect these scores.

#### Intentions to Use Again and Recommend to a Friend

Just over half of reviewers intended to use the app they had chosen again (46/ 83, 55%) and most intended to recommend the chosen app to a friend (67/ 83, 81%). No significant differences were found between app functions and intention to use again (*P*=.52) nor recommend to a friend (*P*=.40). Overall, reviewers indicated that the apps were worth trialling but a few felt improvements or amendments were needed before regular use.

#### Ease of Use Per App

Across the apps, there was a significant positive correlation between ease of use and usefulness (*r*_83_=.45, *P*<.001, one tailed). Most reviewers (65/83, 78%) thought their chosen app was easy or very easy to use. This varied from 100% for two apps (You + Your Diabetes and T1NDA) to 33% (27/83) for another. Reviewers’ felt the easiest to use apps were self-explanatory and simple to understand. The other apps were also considered easy to use but with some suggestions to improve the user-interface.

#### Useful App Features

By app function, the most useful features reported in qualitative feedback were: for data logging apps (1) setting targets and viewing trends, (2) ease of recording and tracking data, and (3) data storage in one mobile location without need for logbook or pen/ paper; for the insulin dose calculation apps (1) simplicity and ease of use, (2) accuracy and trust of calculator, and (3) all in one calculation (carbs and insulin); for the notes/ agenda setting apps (1) the topic prompts to identity and remember what to discuss at appointment, (2) simple layout and ease of use, and (3) ability to document and review notes.

#### The Importance of the Apps Being Created by YPD

Of the 83 reviewers, 34 (41%) responded to additional follow-up. Most of these (n=23, 68%) felt it was important or very important that the apps were created by YPD. In additional comments (91%, 31/34) much importance was placed on app design (not necessarily development) by diabetic peers because of a mutual understanding of the needs, condition and experiences in order for the apps to offer the most accurate features and details. Two reviewers felt that apps created by like-minded people were reassuring (ie, what benefits the developer, benefits the user). A few mentioned the importance of this age group designing the apps from their perspectives; however for other reviewers age was not important as older people with diabetes experience similar issues. A few felt no importance for the apps to be designed by YPD as long as the needs were met, it worked well and looked good, and YPD feedback shaped the design.

## Discussion

### Principal Findings

The most important finding of the Diabetes App Challenge was confirmation of the feasibility of recruiting YPD through an online competition in a relatively short space of time, and with optional support, to develop their own apps to improve preparation for diabetes appointments. We had thought that an online competition would be quicker, more cost effective, and involve larger numbers of YPD, than methods based on a user-panel informing the design of professionally developed app [25,26]. We thought the competition approach might be similar to “hackathons” in rapid collaboration generating impromptu innovation and problem solving [27]. The competition successfully produced six submitted and developed apps: Diabetes Logger, Diabetes Health Tracker, Insulin Calc, cpSlider, T1NDA and You + Your Diabetes. Recruiting larger numbers of YPD to test apps online and provide feedback of their experience was also successful, resulting in 83 completed reviews. Therefore, a competition with online recruitment for design and testing compared very favorably with more expensive and time consuming face-to-face methods.

### Developer Stage

The need for YPD, and not just professionals, to be involved in app design and development was emphasised by the partly unexpected range of apps submitted. Originally, we had not anticipated data recording or insulin dose calculation apps as among the tools to engage YPD in preparation for clinic appointments. Although notes and agenda setting items were featured within most of the submissions, only one of the five other apps (excluding the student example) had consultation agenda setting as a principal function. However, this might be because the YPD who designed these apps considered technical issues to be paramount in being able to engage fully at their appointments. In support of this, some reviewers commented that being able to show the doctor their results on a mobile, and others that helping them deal with the problem of calculating insulin doses, gave them more control and so empowered them in the consultation.

Another significant finding was that the user-developer method we employed was considered important by two-thirds of reviewers. The direct “bottom up” process of peer innovation (ie, what benefits the developer, benefits the user) was valued, although some felt a more “professional” interface was needed.

The study also provided useful information about how to advertise and recruit for a competition. The most effective methods of advertising the competition were university computer science department emails and Diabetes UK online, whereas paid online advertising was considered expensive and ineffective, costing GBP 1700, and resulted in only one potential developer recruited.

A range of unexpected findings, challenges and limitations were also evident in this study. A variety of apps already existed for recording blood glucose, help with carbohydrate and calorie calculations [28], and we had not initially thought that these functions would be considered directly important to agenda setting. We did not specifically ask app developers or reviewers about other apps they may have previously used. At least one of our “drop out” developers withdrew when he considered that his idea had already been developed. In the future, it may be more appropriate for an objective independent review to determine whether specific functions are already met by existing apps. However, we note that similar limitations could affect “hackathons”.

The apps that were produced may also have represented the “art of the possible”, since technical ability was cited as a potential limitation to what could be produced. For example, this may have accounted for a lack of any social media element to the apps. YPD may also have been more adventurous in their ideas if others with greater expertise and resources were to create the apps. Therefore, perhaps professional developer collaborations, or competitions in which YPD just submit ideas for design, rather than actually implement apps, could be the best solution in a future competition.

A probable limitation on the number of apps submitted was the time of year in which the competition took place. The study was scheduled to coincide with the UK summer term and holidays with the intention to “capture” students with available time and interest. However, many students still cited university commitments as reasons for non-participation or withdrawal. Our timescale was also limited by the funding and ethical approval, but a competition run earlier in the year may have attracted greater numbers.

### Reviewer Stage

We successfully recruited a large number of reviewers, and while we failed to reach the target of 200 reviewers in the time available, we think our pilot study clearly confirms the feasibility of this approach. The main time constraint was the requirement to test apps prior to the next booked diabetes clinic appointment. Without this fixed requirement, and by recruiting over a longer period, we would have been able to recruit our target of 200 YPD to test apps.

Diabetes UK and social media platforms Twitter and Facebook were most effective for raising awareness to reviewers. Reviewer participants were self-selected, indicating pre-interest in their diabetes management, yet over a third had previously missed or considered missing a clinic appointment. “Did not attend” rates vary from one clinic to another and over time [29] making it difficult to generalise to the UK population [11]. Future recruitment via clinics might reduce self-selection bias, but another important focus for further research is to determine whether “hard to engage” patients might be more willing to engage in research through popular social media channels than clinics.

Reviewers chose evenly between the 3 main functions of the 6 apps which were suited to different YPD needs for (1) remembering what to ask at appointments, (2) simplifying insulin dose calculations, and (3) easier observation of blood glucose trends. As anticipated in the context of the design specification, notes/ agenda setting apps were perceived as most useful for clinic appointment focus and preparation, in particular, their category prompts for stimulating reflection about areas of concern and reminders to raise issues at the appointment. Despite a large market of diabetes-specific apps (via iTunes and Google Play) none that prioritise this function appeared to be available in 2013 [28]. Some included optional note-making features but did not offer question or category prompts. Previous work suggested that written pre-clinic check sheets may improve question-asking [30] and appointment satisfaction [31] but this has yet to be fully explored using mobile technology.

Lastly, the online user-review method enabled larger scale remote recruitment, comparable to online usability testing, a common method of assessing the user experience of websites and apps [32]. The anonymity of online feedback can also enable expression of opinion from those hard to reach by other face-to-face means [33]. While face-to-face evaluation might have allowed more detailed feedback, the speed and cost of online research methods made them very attractive in this research.

### Conclusions

This pilot study confirmed the feasibility of engaging YPD in a competition to design and test apps to enhance preparation for clinic appointments. A range of needs were identified in the apps that were designed and some of our preconceptions about likely app functions were challenged. This study strongly supports the idea that YPD should be involved in designing apps for use by YPD, but it may be more appropriate for the primary role of the patients to be to advise on design rather than implementation. It will be particularly important to determine whether competitive app design and evaluation could engage more hard to reach YPD who are at highest risk from poor control of diabetes, as opposed to more self-selected enthusiasts who engaged with this project.

## Abbreviations

YPD: young people with diabetes
NHS: National Health Service
UK: United Kingdom

## References

[1] Chisolm DJ, Johnson LD, McAlearney AS (2011). What makes teens start using and keep using health information web sites? A mixed model analysis of teens with chronic illnesses. Telemed J E Health.

[2] Isomursu P, Isomursu M, Leinonen E (2005). Association for Consumer Research. User involvement of different target groups in a mobile context. European Advances in Consumer Research.

[3] Kortuem G, Kawsar F (2010). Market-based User Innovation for the Internet of Things. Internet of Things 2010 Conference (IoT-2010).

[4] Boudreau KJ, Lacetera N, Lakhani KR (2011). Incentives and Problem Uncertainty in Innovation Contests: An Empirical Analysis. Management Science.

[5] Stack Exchange (2012). Apptivate.

[6] Department of Health (2011). Call for ideas for new health apps and maps.

[7] Department of Health (2011). Read all about your favourite health apps and ideas.

[8] Diabetes UK (2012). Diabetes in the UK: key statistics on diabetes.

[9] Nathan DM, Zinman B, Cleary PA, Backlund JY, Genuth S, Miller R, Orchard TJ, Diabetes ControlComplications Trial/Epidemiology of Diabetes InterventionsComplications (DCCT/EDIC) Research Group (2009). Modern-day clinical course of type 1 diabetes mellitus after 30 years' duration: the diabetes control and complications trial/epidemiology of diabetes interventions and complications and Pittsburgh epidemiology of diabetes complications experience (1983-2005). Arch Intern Med.

[10] Secrest AM, Becker DJ, Kelsey SF, LaPorte RE, Orchard TJ (2010). All-cause mortality trends in a large population-based cohort with long-standing childhood-onset type 1 diabetes: the Allegheny County type 1 diabetes registry. Diabetes Care.

[11] Snow R, Fulop N (2012). Understanding issues associated with attending a young adult diabetes clinic: a case study. Diabet Med.

[12] Channon S, Hambly H, Robling M, Bennert K, Gregory JW, DEPICTED Study Team (2010). Meeting the psychosocial needs of children with diabetes within routine clinical practice. Diabet Med.

[13] Bensing J (2000). Bridging the gap. The separate worlds of evidence-based medicine and patient-centered medicine. Patient Educ Couns.

[14] Allen D, Gregory J (2009). The transition from children's to adult diabetes services: understanding the 'problem'. Diabet Med.

[15] Cafazzo JA, Casselman M, Hamming N, Katzman DK, Palmert MR (2012). Design of an mHealth app for the self-management of adolescent type 1 diabetes: a pilot study. J Med Internet Res.

[16] Frost J, Anderson R, Argyle C, Daly M, Harris-Golesworthy F, Harris J, Gibson A, Ingram W, Pinkney J, Ukoumunne Oc, Vaidya B, Vickery J, Britten N (2013). A pilot randomised controlled trial of a preconsultation web-based intervention to improve the care quality and clinical outcomes of diabetes outpatients (DIAT). BMJ Open.

[17] Youen S (2011). You + Your Diabetes.

[18] Stemler S (2001). Practical Assessment, Research & Evaluation, 7(17).

[19] Hsieh HF, Shannon SE (2005). Three approaches to qualitative content analysis. Qual Health Res.

[20] Henagulph C, Fisher S (2012). Diabetes Logger.

[21] Hearty M, Leonard P, Trever J, Ali V (2012). Diabetes Health Tracker.

[22] Chanter W (2012). T1NDA.

[23] Ahmed W, Nagpal J, Bandhu.

[24] Pashley D, Walton S, McKeown D (2012). cpSlider.

[25] McCurdie T, Taneva S, Casselman M, Yeung M, McDaniel C, Ho W, Cafazzo J (2012). Horizons; Fall.

[26] Goodman-Deane J, Langdon P, Clarkson J (2010). Key influences on the user-centred design process. J. of Eng. Design.

[27] Petti MA (2013). National Conference on Health Communication, Marketing, and Media.

[28] Jones RB, Cleverly L, Hammersley S, Ashurst E, Pinkney J (2013). Journal of Diabetes Nursing.

[29] Masding M, Klejdys S, MacHugh B, Gale S, Brown A, McAulay A (2010). Non-attendance at a diabetes transitional clinic and glycaemic control. Pract Diab Int.

[30] Glynne-Jones R, Ostler P, Lumley-Graybow S, Chait I, Hughes R, Grainger J, Leverton TJ (2006). Can I look at my list? An evaluation of a 'prompt sheet' within an oncology outpatient clinic. Clin Oncol (R Coll Radiol).

[31] Kinnersley P, Edwards A, Hood K, Cadbury N, Ryan R, Prout H, Owen D, Macbeth F, Butow P, Butler C (2007). Interventions before consultations for helping patients address their information needs. Cochrane Database Syst Rev.

[32] Albert W, Tedesco D, Tullis T (2009). Beyond the Usability Lab: Conducting Large-scale Online User Experience Studies.

[33] Tates K, Zwaanswijk M, Otten R, van Dulmen S, Hoogerbrugge PM, Kamps WA, Bensing JM (2009). Online focus groups as a tool to collect data in hard-to-include populations: examples from paediatric oncology. BMC Med Res Methodol.

